# A case of seronegative anti-glomerular basement membrane disease with linear IgG deposition

**DOI:** 10.1080/0886022X.2022.2146314

**Published:** 2022-11-15

**Authors:** Ning Zhuo, Gang Wang, Panai Song, Yao Liu, Shuqing Li, Yinghong Liu

**Affiliations:** a Department of Nephrology, The Second Xiangya Hospital of Central South University, Changsha, China; b Department of Rheumatology and Immunology, The Second Affiliated Hospital of Soochow University, Suzhou, China

Dear Editor,

A 29-year-old male worker presented to the hospital with recurrent facial and bilateral lower limb edema for 3 months. He presented with a history of smoking for over 10 years and had no history of hypertension, diabetes mellitus, cardiovascular disease, and no family history of genetic disorders or kidney disease. Laboratory tests on admission showed serum creatinine 127 μmol/L, hemoglobin 91 g/L, urine protein 6 g/L, urine occult blood (++), albumin 19.94 g/L, triglycerides 2.85 mmol/L, total cholesterol 7.39 mmol/L, with normal complement C3 and C4. Negative serology for anti-glomerular basement membrane (anti-GBM) antibody, anti-phospholipase A2 receptor (PLA2R) antibody, anti-nuclear antibody, anti-double stranded DNA, pANCA and cANCA, serum and urine immunofixation electrophoresis, human immunodeficiency virus (HIV), hepatitis B virus (HBV), and hepatitis C virus (HCV). Renal ultrasound showed enlargement of both kidneys.

Subsequent renal biopsy pathology included: light microscopy showing moderate to severe mesangial proliferative glomerulonephritis with focal segmental necrosis and crescent formation, along with acute tubulointerstitial nephritis. Immunofluorescence revealed linear deposition of IgG (+++) in capillary collaterals, C3 (−), C1q (−), Fib (−), ALB (−), κ(+), and lambda (++). Congo red staining was negative. Electron microscopy revealed segmental thickening of the glomerular basement membrane with wide fusion of foot processes, and a small amount of low-density electron-dense deposits, as well as disorganized fibrillar-like structures, were seen in individual mesangial areas. Immunoelectron microscopy showed negative DNAJB9 staining (Figure 1). A final diagnosis of seronegative anti-GBM disease was made.

**Figure 1.**
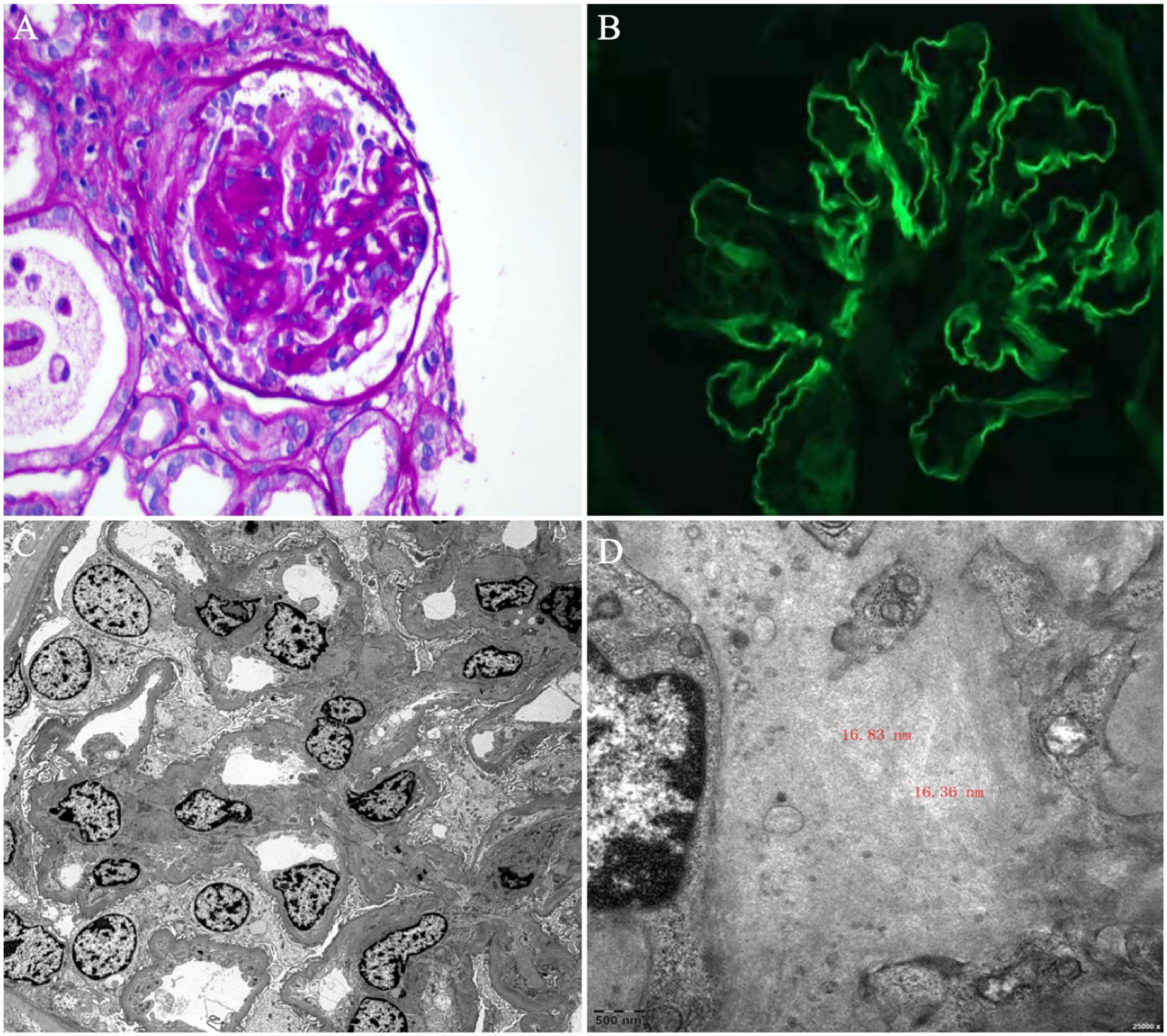
(A) Light microscopy shows the cellular crescent formation and vacuolar degeneration of some epithelial cells of the renal tubules (PAS staining, magnification × 400). (B) Immunofluorescence shows linear deposition of IgG (+++) in capillary collaterals. (C) Electron microscopy shows segmental thickening of the glomerular basement membrane with an extensive fusion of the foot process and a small amount of low electron-dense material. (D) Electron microscopy shows disorganized fibrous structures in the mesangial region.

He then received methylprednisolone 500 mg/day pulse therapy for three days and cyclophosphamide therapy followed by hormone taper over two months. Since the patient was immunocompromised and had a co-infection of the lungs, the treatment regimen was adjusted to moderate doses of hormones combined with four plasma exchanges and prophylactic use of compounded sulfamethoxazole. However, the circulating volume load remained heavy, and he was eventually discharged after improving on hemodialysis. Since then the patient has been followed up regularly and at the most recent follow-up, the patient was stable with a blood creatinine of 258 μmol/L, urinary occult blood (+++), and urinary protein (+++). The timeline of serum creatinine changes over time and treatment, in this case, is shown in the figure (Figure 2).

**Figure 2.**
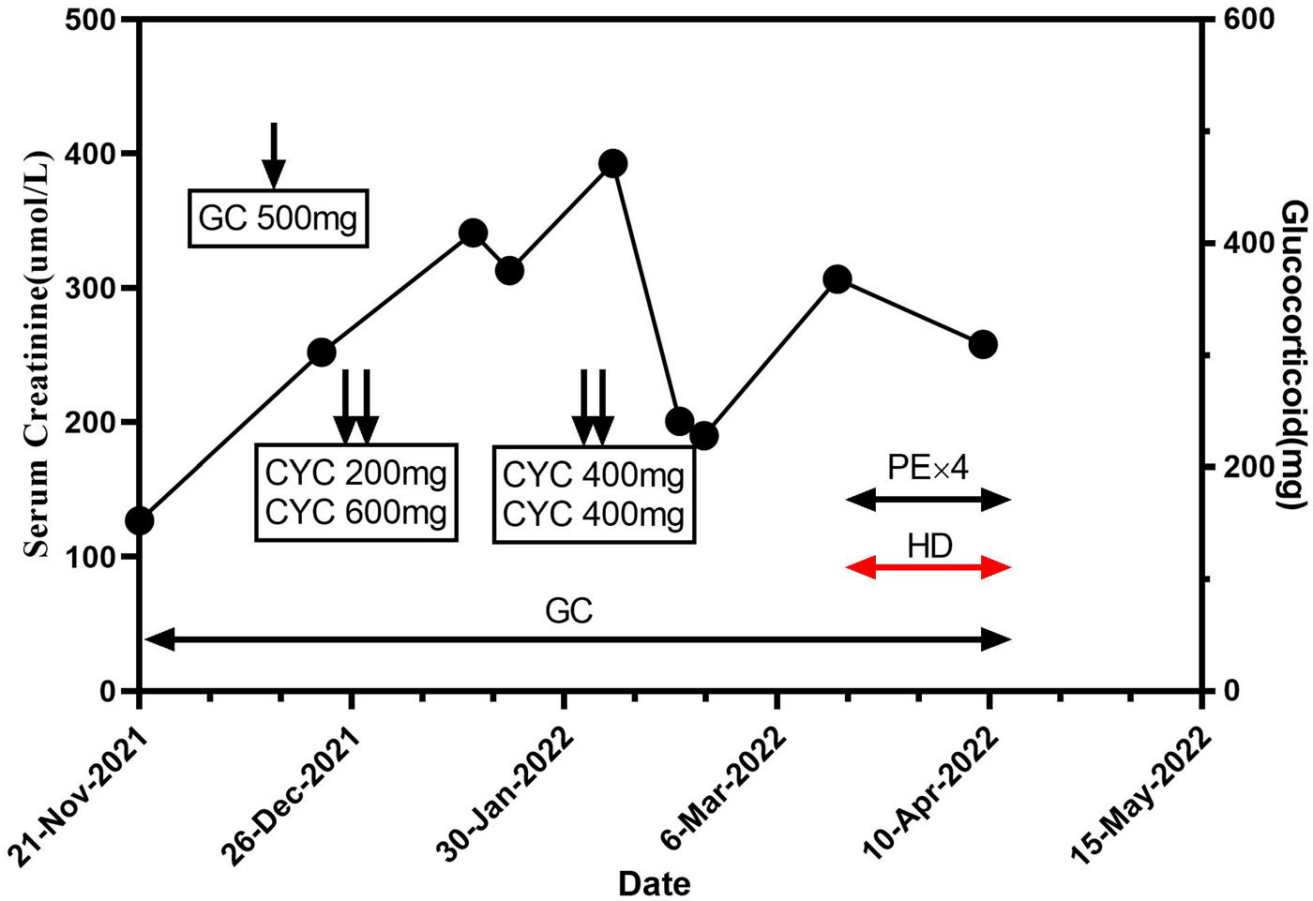
Timeline of serum creatinine changes over time and treatment in this case. Abbreviations: GC: glucocorticoids; CYC: cyclophosphamide; PE: plasma exchange; HD: hemodialysis.

Anti-GBM disease is a rare autoimmune disease caused by anti-GBM antibodies to the noncollagenous structural domain (NC1) of the alpha3 (α3) chain of type IV collagen, a well-characterized autoantigen in the basement membrane of the kidney or lung that causes rapidly progressive glomerulonephritis or pulmonary hemorrhage [1]. The main diagnostic criteria for this disease are the detection of anti-GBM antibodies in the circulation and renal immunofluorescence showing linear deposition of immunoglobulin G along the glomerular basement membrane [1]. In this case study, we report a seronegative anti-GBM disease. Consistent with the typical anti-GBM disease, the renal pathology of this patient showed a linear pattern of antibody deposition in the glomerular basement membrane. However, mesangial proliferative glomerulonephritis and tubulointerstitial nephritis were also present, which is uncommon in typical anti-GBM disease. Nasr et al. described a series of morphologic features of the atypical anti-GBM disease, including capillary intimal proliferative glomerulonephritis, mesangial proliferative changes, and focal segmental sclerosis. The tubular interstitium may show varying degrees of fibrosis and tubular atrophy, with or without inflammatory changes. In immunofluorescence, monoclonal IgG deposition is present in 50% of cases, with IgA or IgM deposition in a minority of cases [2].

The renal pathology in our case showed linear deposition of IgG in capillary collaterals and immunofluorescence of C3 (−), C1q (−), Fib (−), ALB (−), κ(+), lambda (+++), which could exclude diabetic nephropathy, which manifests as albumin deposition. Notably, we observed a small amount of low-density electron-dense deposits in the mesangial region under electron microscopy, with a fiber diameter of about 6–16 nm.

Previous studies have shown that 33 of 59 (55.9%) seronegative anti-GBM diseases revealed dense deposits on electron microscopy [3]. Specific structural deposits were also seen in fibrous glomerulonephritis, amyloidosis, and immunotactoid glomerulopathy. Negative Congo red staining excludes renal amyloidosis. In immunotactoid glomerulopathy, electron microscopy revealed characteristic microtubular deposits with a diameter of 14–60 nm, hollow cores, frequent parallel alignment, and a predominant distribution outside of the lamina densa of the glomerular basement membrane, which were not found in our case [4]. Fibrillary glomerulonephritis is an immune-complex glomerulonephritis with mesangial hyperplasia as the most common type of lesion. Fluorescence microscopy of fibrillary glomerulonephritis shows mainly IgG and C3 staining in the mesangial region, with weak capillary wall staining, sometimes granular and irregular. Electron microscopy reveals randomly distributed straight fibrils with a diameter of 12–24 nm [5]. Studies have shown that serum DNAJB9 levels can accurately predict fibrillary glomerulonephritis with a sensitivity of 67%, specificity of 98%, and positive and negative predictive values of 89% and 95%, respectively [6]. It is recommended to refine the laser microdissection/mass spectrometry analysis. Unfortunately, this is not yet available at our institution and has not been completed.

There are several reasons why patients have linear IgG deposits without having positive test results for circulating antibodies. Our case was tested for anti-GBM antibodies in the peripheral circulation a total of two times, with an interval of more than two months, and both results were negative. It is unlikely that negative anti-GBM antibodies are due to low titers of circulating antibodies or a short antibody half-life. A rare form of anti-GBM glomerulonephritis mediated by IgA autoantibodies has been described in 11 patients in one study. The target of the patient’s IgA autoantibodies was identified as a novel GBM antigen [7]. A renal pathology of 60 patients with atypical anti-GBM disease revealed that all patients exhibited visible linear deposits of IgG along GBM, with IgG1 being the predominant subclass (45.8%), followed by IgG2 (35.6%), IgG4 (18.6%) and IgG3 (11.9%) [3]. Therefore, we speculate that antibodies to nontraditional GBM epitopes, non-IgG anti-GBM antibodies, or different IgG subclasses, such as IgG4 instead of IgG1, may be present in our cases. This suggests that antibodies with low affinity can be determined by higher sensitive assays such as western blot and biosensor experiments [8]. Serum anti-GBM antibodies can be detected by indirect immunofluorescence using normal kidney tissue [3].

## Data Availability

The original contributions presented in the study are included in the article. Further inquiries can be directed to the corresponding author.
